# Wnt-Signaling Inhibitor Wnt-C59 Suppresses the Cytokine Upregulation in Multiple Organs of Lipopolysaccharide-Induced Endotoxemic Mice via Reducing the Interaction between β-Catenin and NF-κB

**DOI:** 10.3390/ijms22126249

**Published:** 2021-06-10

**Authors:** Jaewoong Jang, Jaewon Song, Inae Sim, Young V. Kwon, Yoosik Yoon

**Affiliations:** 1 Department of Microbiology, College of Medicine, Chung-Ang University, Seoul 06974, Korea; jjw4207@naver.com (J.J.); s536142@naver.com (J.S.); siminae@naver.com (I.S.); 2 Department of Biochemistry, University of Washington, Seattle, WA 98195, USA; ykwon7@uw.edu

**Keywords:** Wnt-C59, Wnt, β-catenin, NF-κB, cytokine, endotoxemia

## Abstract

Sepsis is characterized by multiple-organ dysfunction caused by the dysregulated host response to infection. Until now, however, the role of the Wnt signaling has not been fully characterized in multiple organs during sepsis. This study assessed the suppressive effect of a Wnt signaling inhibitor, Wnt-C59, in the kidney, lung, and liver of lipopolysaccharide-induced endotoxemic mice, serving as an animal model of sepsis. We found that Wnt-C59 elevated the survival rate of these mice and decreased their plasma levels of proinflammatory cytokines and organ-damage biomarkers, such as BUN, ALT, and AST. The Wnt/β-catenin and NF-κB pathways were stimulated and proinflammatory cytokines were upregulated in the kidney, lung, and liver of endotoxemic mice. Wnt-C59, as a Wnt signaling inhibitor, inhibited the Wnt/β-catenin pathway, and its interaction with the NF-κB pathway, which resulted in the inhibition of NF-κB activity and proinflammatory cytokine expression. In multiple organs of endotoxemic mice, Wnt-C59 significantly reduced the β-catenin level and interaction with NF-κB. Our findings suggest that the anti-endotoxemic effect of Wnt-C59 is mediated via reducing the interaction between β-catenin and NF-κB, consequently suppressing the associated cytokine upregulation in multiple organs. Thus, Wnt-C59 may be useful for the suppression of the multiple-organ dysfunction during sepsis.

## 1. Introduction

The Wnt pathway is involved in various physiological and pathological processes, including embryonic development, tissue homeostasis, and malignant transformation [1]. Until now, most studies on the Wnt pathway have focused on its role in cancer. For example, dysregulation of the Wnt pathway has been observed in various types of cancers in humans [2], and many Wnt-signaling inhibitors have been developed for the treatment of Wnt-driven cancers [3]. Wnt-C59, which inhibits the palmitoylation and secretion of Wnt, has been developed as an anti-cancer drug candidate and shows anti-tumor activity in mouse models of Wnt-driven cancers, including mammary cancer [4] and nasopharyngeal carcinoma [5]. Wnt-C59 also inhibits the three-dimensional sphere formation of cancer stem cells by modulating the tumor microenvironment [5].

Sepsis is the most common cause of death in intensive care units, with no effective cure currently available, and there is a high need for an effective antisepsis treatment [6]. Recently, it has been reported that the Wnt signaling is involved in sepsis [7]. For example, a study reported significantly upregulated WNT5A levels in the serum of a sepsis patient [8] and in the lung biopsy from a patient who died of sepsis, compared with the healthy-control levels [9]. Additionally, the levels of multiple WNT ligands are elevated in the peripheral blood of patients with septic shock and in the spleen of endotoxemic mice [10]. A Wnt signaling inhibitor, iCRT3, has been reported to suppress lung injury in a murine model of sepsis [11]. The elucidation of the involvement of the Wnt pathway in sepsis has presented this pathway as a potential target for antisepsis therapy.

Sepsis is defined as a life-threatening organ dysfunction caused by the dysregulated host response to an infection [12]. Organ dysfunction during sepsis occurs in the kidney, lung, liver, heart, central nervous system, and hematological system, and this multiple-organ dysfunction is the major cause of sepsis-induced death [13]. As sepsis is characterized by multiple-organ failure [12], the Wnt signaling during sepsis should be characterized in multiple organs. Until now, however, no study has fully analyzed in multiple organs whether the Wnt signaling is activated during sepsis and whether the modulation of this pathway can rescue sepsis-associated multiple organ dysfunction.

Endotoxemia is a type of sepsis induced by endotoxins, the major virulence factor of Gram-negative bacteria [14]. The incidence of endotoxemia among patients with sepsis, particularly those infected with Gram-negative bacteria, can be as high as 82% [15]. Therefore, endotoxemic mice have been frequently used as a sepsis animal model [16,17,18]. In this study, we analyzed the Wnt signaling and assessed for the effect of Wnt-C59 in multiple organs, such as the kidney, lung, and liver, of lipopolysaccharide (LPS)-induced endotoxemic mice.

## 2. Results

### 2.1. Wnt-C59 Elevated the Survival Rate and Decreased the Plasma Levels of Proinflammatory Cytokines and Organ-Damage Biomarkers in Endotoxemic Mice

**Figure 1:**
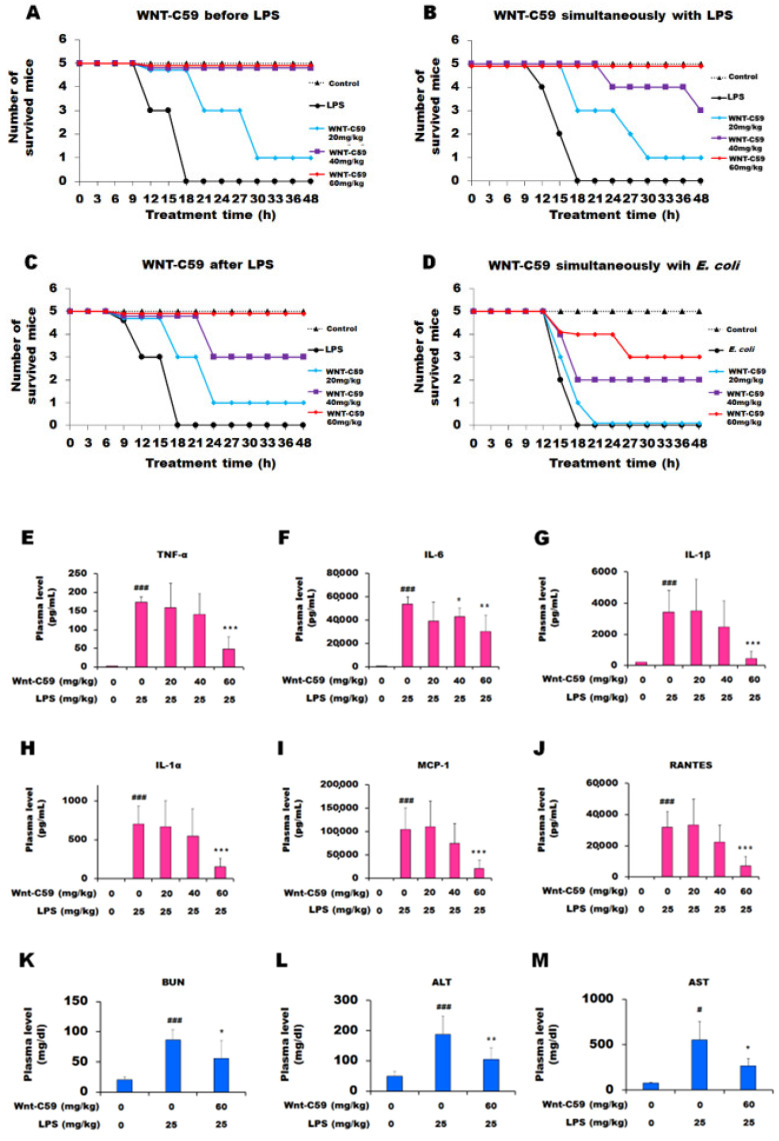
Wnt-C59 reduced the lethality and plasma levels of proinflammatory cytokines and organ-damage biomarkers in endotoxemic mice. (**A**–**D**) Wnt-C59 suppressed the lethality of endotoxemic mice (*n* = 5). C57BL/6 mice were i. p. injected with 0, 20, 40, or 60 mg/kg of Wnt-C59 (**A**) 2 h before, (**B**) simultaneously with, or (**C**) 1 h after injecting 25 mg/kg of lipopolysaccharide (LPS). (**D**) Wnt-C59 at 0, 20, 40, or 60 mg/kg was i. p. injected simultaneously with 10^11^ viable *E. coli* cells. The control group was injected with saline. (**E**–**J**) Plasma cytokine concentrations were measured using a Luminex assay (*n* = 7). (**K**–**M**) The levels of BUN, a kidney-damage biomarker, as well as ALT and AST, liver-damage biomarkers, were measured using a veterinary biochemistry analyzer (*n* = 7), respectively. * *p* < 0.05, ** *p* < 0.01, and *** *p* < 0.001 compared with the group injected with 25 mg/kg of LPS. # *p* < 0.05 and ### *p* < 0.001 compared with the control group (unpaired *t*-test). LPS: lipopolysaccharide; *E. coli*: *Escherichia coli*; BUN: blood urea nitrogen; ALT: alanine aminotransferase; AST: aspartate aminotransferase.

In the mice that received LPS alone, the survival rate was 0%. However, the mice injected with 20 mg/kg of Wnt-C59 2 h before LPS administration showed a survival rate of 20%, and those treated with 40 or 60 mg/kg of Wnt-C59 had a survival rate of 100% (5 mice per group) (Figure 1A). Wnt-C59 elevated the survival rate of LPS-induced endotoxemic mice when injected simultaneously with LPS (Figure 1B) or 1 h after LPS injection (Figure 1C). Moreover, Wnt-C59 elevated the survival rate when injected simultaneously with 10^11^ viable *E. coli* cells (Figure 1D). These findings clearly demonstrate that the endotoxemic death caused by LPS or bacteria was suppressed by Wnt-C59 in a dose-dependent manner.

Under the healthy state, cytokine expression is limited to modulate the inflammatory response; however, endotoxemia or sepsis causes excessive cytokine upregulation, a phenomenon named a “cytokine storm.” A cytokine storm is caused by the auto-amplification of cytokine expression, consequently resulting in the failure of multiple organs and, finally, death [19,20]. We found that plasma levels of proinflammatory cytokines, such as TNF-α, IL-6, IL-1β, IL-1α, MCP-1, and RANTES, were drastically increased in the LPS-induced endotoxemic mice compared with those of the control mice, indicative of the cytokine storm (Figure 1E–J). Wnt-C59 treatment significantly reduced the plasma cytokine levels in a dose-dependent manner. The dose of 60 mg/kg showed the best protection against the LPS-induced lethality and cytokine storm, and thus further experiments were conducted at this dose.

Plasma levels of the biomarkers of kidney and liver damage, such as BUN, ALT, and AST, were elevated in the endotoxemic mice compared with the levels in the control mice, demonstrating multiple-organ damage during endotoxemia. Wnt-C59 significantly suppressed the LPS-induced upregulation of these biomarkers, thus exhibiting a protective effect against organ damage (Figure 1K–M).

### 2.2. Wnt-C59 Suppressed the Cytokine Upregulation and NF-κB Activity in Multiple Organs of Endotoxemic Mice

**Figure 2:**
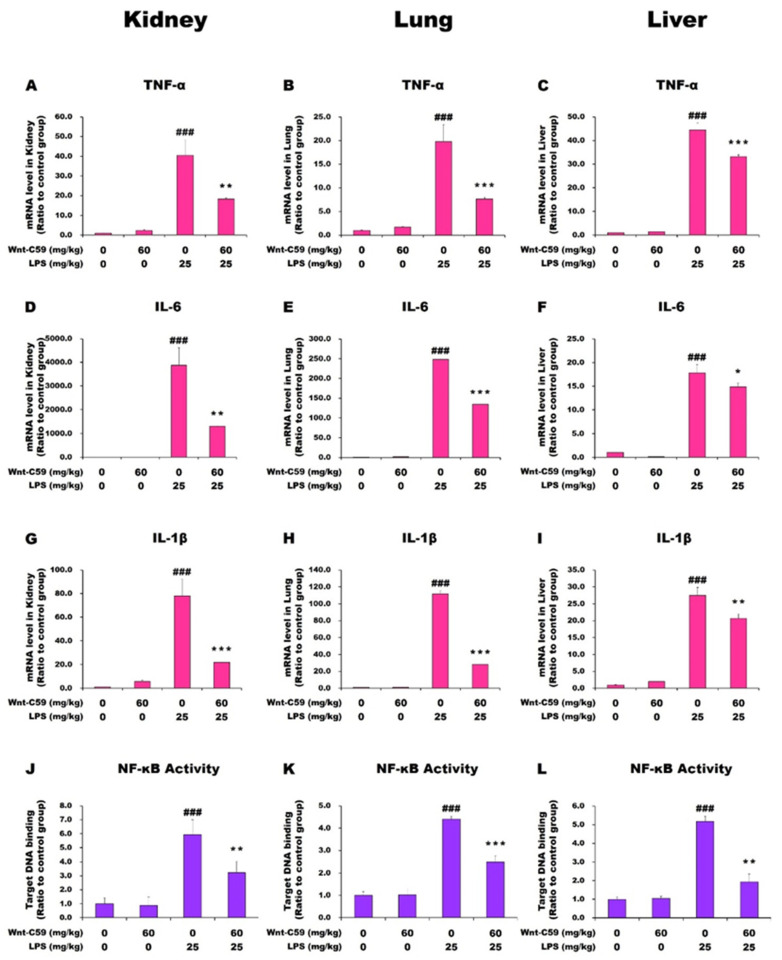
Wnt-C59 suppressed the cytokine upregulation and NF-κB activity in multiple organs of endotoxemic mice. C57BL/6 mice were i. p. injected with 0 or 60 mg/kg of Wnt-C59 and then with 0 or 25 mg/kg of lipopolysaccharide (LPS) after 2 h. (**A**–**I**) The cytokine mRNA levels in the kidney, lung, and liver were quantified via reverse transcription–quantitative polymerase chain reaction (*n* = 4). (**J**–**L**) The target-DNA binding activity of NF-κB in the kidney, lung, and liver was measured using ELISA (*n* = 4). * *p* < 0.05, ** *p* < 0.01, and *** *p* < 0.001 compared with the group injected with 25 mg/kg of LPS. ### *p* < 0.001 compared with the control group (unpaired *t*-test).

The levels of cytokine expression in multiple organs, including the kidney, lung, and liver, were measured via reverse transcription–quantitative polymerase chain reaction (RT-qPCR). Experimental mice were injected with 0 or 60 mg/kg of Wnt-C59 2 h before LPS injection, while control mice were injected with saline. In the kidney, lung, and liver of the LPS-induced endotoxemic mice, *TNF-α*, *IL-6*, and *IL-1β* mRNA levels were markedly increased compared with the levels in the control mice. Wnt-C59 treatment significantly suppressed the upregulation of cytokine mRNA levels in LPS-stimulated mice but had no effect in unstimulated mice (Figure 2A–I). The *MCP-1* and *IL-1α* mRNA levels showed identical patterns with those of the above-mentioned cytokines (Figure S1). These data showed that proinflammatory cytokines were upregulated in multiple organs of the endotoxemic mice, but this phenotype was significantly suppressed by Wnt-C59 treatment.

It is well-known that NF-κB is a major proinflammatory transcription factor that binds to its target sequence in the promoter of many cytokine genes to activate their expression [21]. The target sequence-binding activity of NF-κB showed similar patterns with those of the proinflammatory cytokine mRNA levels in multiple organs (Figure 2J–L). Our data showed that the elevation in NF-κB activity is the cause of cytokine upregulation in multiple organs of endotoxemic mice, and the suppressive effect of Wnt-C59 on the cytokine upregulation results from the suppression of the NF-κB activity.

### 2.3. The Wnt/β-Catenin and NF-κB Pathways Were Activated in Multiple Organs of Endotoxemic Mice, and Wnt-C59 Suppressed Both Pathways

**Figure 3:**
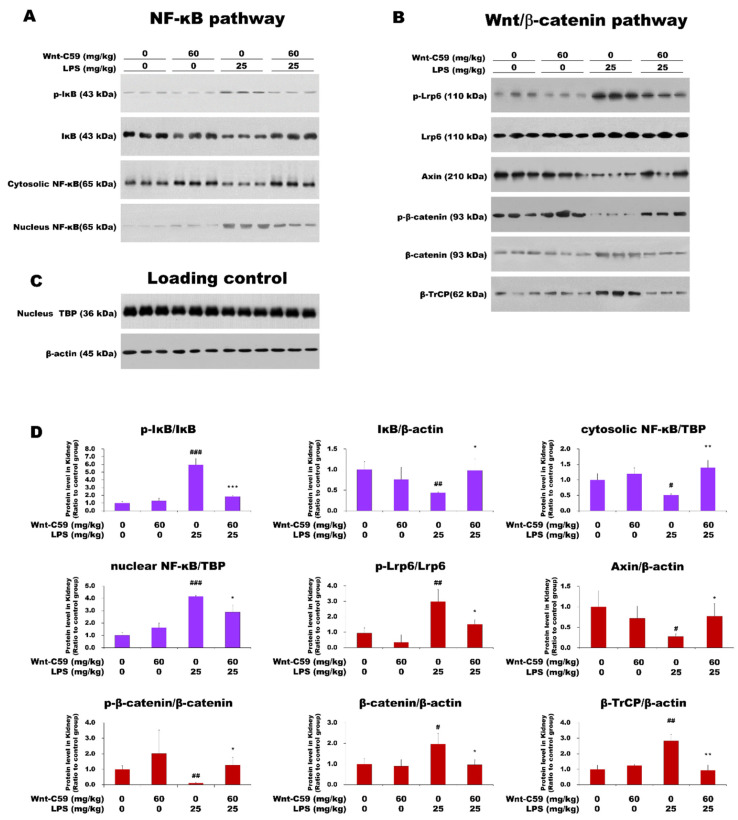
Wnt-C59 suppressed both the NF-κB and Wnt/β-catenin pathways in the kidney. C57BL/6 mice were i. p. injected with 0 or 60 mg/kg of Wnt-C59 and then with 0 or 25 mg/kg of lipopolysaccharide (LPS) after 2 h. (**A**) To measure the levels of the proteins involved in the NF-κB pathway, Western blotting was conducted using kidney protein extract from the endotoxemic mice (*n* = 3). (**B**) The levels of the proteins involved in the Wnt/β-catenin pathway were evaluated via Western blotting using kidney protein extract (*n* = 3). (**C**) β-Actin and TBP were used as loading controls for total and nuclear lysates, respectively. (**D**) The Western-blot band intensities of the members of the NF-κB and Wnt/β-catenin pathways are shown in violet and red, respectively. The target band intensities were quantified using ImageJ (NIH, Bethesda, MD, USA) and were normalized to the band intensities of the loading controls. The data show the average ± standard deviation (*n* = 3). * *p* < 0.05, ** *p* < 0.01, and *** *p* < 0.001 compared with the group injected with 25 mg/kg of LPS. # *p* < 0.05, ## *p* < 0.01, and ### *p* < 0.001 compared with the control group (unpaired *t*-test). TBP: TATA-box–binding protein.

As the target-DNA binding activity of NF-κB was found to be suppressed by Wnt-C59 (Figure 2J–L), the effect of Wnt-C59 on the NF-κB pathway was analyzed in multiple organs via Western blotting. Our data showed that the LPS-induced activation of the NF-κB pathway was suppressed by Wnt-C59 in the kidney of endotoxemic mice (Figure 3A,D); the level of phospho-IκB (p-IκB) was reduced and the IκB level was increased by Wnt-C59. The cytoplasmic level of NF-κB was increased by Wnt-C59 treatment, whereas the nuclear level was reduced. These observations indicate that the nuclear translocation of NF-κB was suppressed by Wnt-C59. Of note, Wnt-C59 treatment alone had no effect on the NF-κB pathway in mice without LPS stimulation, compared with the control levels.

**Figure 4:**
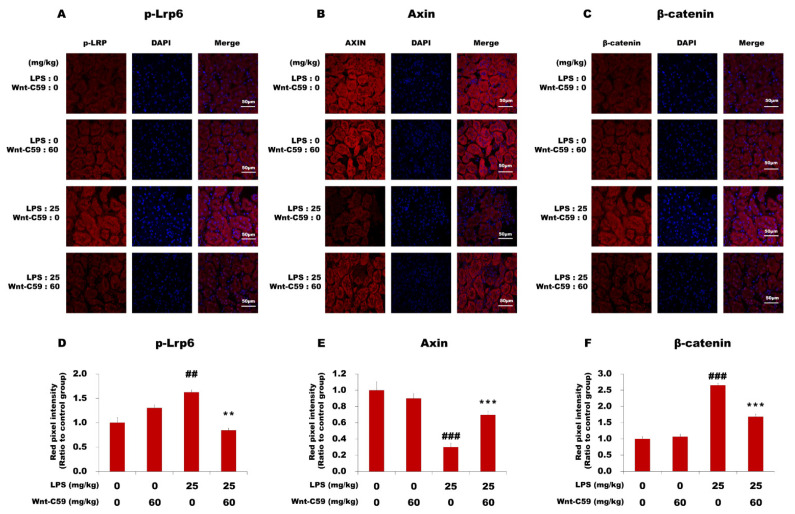
Immunofluorescence analysis of the major proteins in the Wnt/β-catenin pathway in the kidney. C57BL/6 mice were i. p. injected with 0 or 60 mg/kg of Wnt-C59 and then with 0 or 25 mg/kg of lipopolysaccharide (LPS) after 2 h. (**A**–**C**) Confocal microscopic images of kidney sections immunostained for p-Lrp6, Axin, or β-catenin were taken at 100× magnification, respectively. A white scale bar of 50 μm is shown in each image. (**D**–**F**) The fluorescence signals in the images were quantified using ImageJ (NIH, Bethesda, MD, USA). ** *p* < 0.01 and *** *p* < 0.001 compared with the group injected with 25 mg/kg of LPS. ## *p* < 0.01 and ### *p* < 0.001 compared with the control group (unpaired *t*-test).

Using the same protein samples used for the Western blotting analysis of the NF-κB pathway, we analyzed the Wnt/β-catenin pathway (Figure 3B,D). Our data showed that the Wnt/β-catenin pathway was activated in the kidney during endotoxemia, but this phenotype was suppressed by Wnt-C59 treatment. The level of phospho-Lrp6 (p-Lrp6), which represents the active state of the Wnt signaling, was reduced by Wnt-C59, whereas that of Axin, a negative regulator of the Wnt signaling, was increased. The level of phospho–β-catenin (p–β-catenin) was increased, whereas that of β-catenin was reduced by Wnt-C59. β-TrCP, a protein product of one of the target genes induced by β-catenin [22], was also downregulated by Wnt-C59. The results from immunofluorescence microscopy analyses of the kidney confirmed the activation of the Wnt/β-catenin pathway during endotoxemia and the Wnt-C59–induced inhibition (Figure 4). The immunofluorescence intensities of p-Lrp6 and β-catenin were elevated and that of Axin was reduced during endotoxemia, which were suppressed by Wnt-C59. The analyses of the lung and liver tissues via Western blotting (Figures S2 and S3) and immunofluorescence microscopy (Figures S4 and S5) confirmed that both the NF-κB and Wnt/β-catenin pathways were activated in multiple organs of the endotoxemic mice, and Wnt-C59 suppressed both pathways.

Interestingly, Wnt-C59, a Wnt signaling inhibitor, exerted significant suppressive effects on not only the Wnt/β-catenin pathway but also the NF-κB pathway, suggesting a possible connection between the two pathways in multiple organs of endotoxemic mice.

### 2.4. β-Catenin and NF-κB Interact in Multiple Organs of Endotoxemic Mice, Which Was Suppressed by Wnt-C59

**Figure 5:**
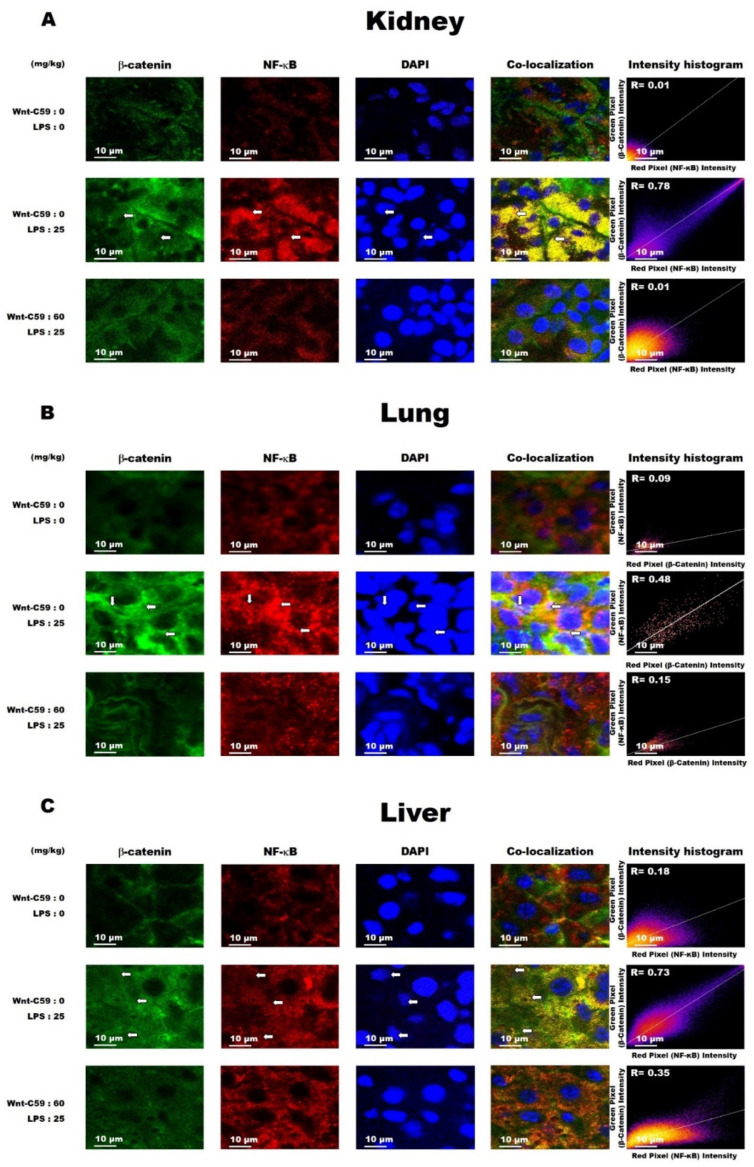
Wnt-C59 suppressed the co-localization of β-catenin and NF-κB in multiple organs of endotoxemic mice. Mice were i. p. injected with 0 or 60 mg/kg of Wnt-C59 and then with 25 mg/kg of lipopolysaccharide after 2 h. The control group was injected with saline. The interaction of β-catenin and NF-κB was measured based on their degree of co-localization, as assessed via immunofluorescence. Immunofluorescence images of the (**A**) kidney, (**B**) lung, and (**C**) liver show the signals for β-catenin (green), NF-κB (red), and DAPI for nuclei (blue). Co-localization is indicated by the yellow color; white arrows show nuclei with the co-localization signals. The DAPI signal was intensified to show the exact locations of the nuclei, but the other images are unmodified. All the images were taken at 600× magnification. The degree of co-localization was evaluated using Pearson’s correlation coefficient (*R*), obtained using ImageJ.

β-Catenin is the executor protein of the Wnt signaling, and NF-κB is the major proinflammatory transcription factor responsible for cytokine expression. To assess for a cross-communication between the Wnt/β-catenin and NF-κB pathways, we performed co-localization analyses to evaluate whether β-catenin interacted with NF-κB in multiple organs of the endotoxemic mice. Our data showed that the co-localization of β-catenin and NF-κB in the kidney was low in the control mice (*R* = 0.01) but was elevated in LPS-induced endotoxemic mice (*R* = 0.78), and this LPS-induced phenotype was inhibited by Wnt-C59 (*R* = 0.01) (Figure 5A). The degrees of β-catenin and NF-κB co-localization in the lung and liver were also elevated by LPS, but this effect was suppressed by Wnt-C59 treatment (Figure 5B,C). The suppressive effect of Wnt-C59 on the interaction between β-catenin and NF-κB was confirmed through co-immunoprecipitation analyses, with results consistent with those derived from the co-localization assays (Figure S6). Our findings are supported by previous reports indicating that β-catenin physically interacts with NF-κB and, consequently, the transcription of NF-κB target genes is enhanced [23], and disrupting this interaction reduces the transcriptional activity of NF-κB [24]. The interaction of β-catenin with NF-κB presumably generates a cross-communication between the Wnt/β-catenin and NF-κB pathways in multiple organs that causes the cytokine upregulation, which was significantly suppressed by Wnt-C59.

## 3. Discussion

In this study, we showed the activation of the Wnt/β-catenin pathway in the kidney, lung, and liver of endotoxemic mice (Figure 3B and Figure 4, and Figures S2–S5). To our knowledge, this is the first report on the activation of the Wnt signaling in multiple organs during sepsis. The Wnt signaling inhibitor Wnt-C59 reduced the organ levels of β-catenin (Figure 4C, Figures S4C and S5C), thus decreasing the interaction of β-catenin with NF-κB (Figure 5 and Figure S6), whereby the transcriptional activity of NF-κB was reduced (Figure 2J–K). These events provide a plausible mechanistic explanation to the suppressive effect of Wnt-C59 on the cytokine storm in multiple organs (Figure 2A–I) and blood (Figure 1E–J), whereby the sepsis-related organ dysfunction (Figure 1K–M) and consequent death (Figure 1A–D) are suppressed.

It has been reported that the high activity of the endotoxin (LPS) is associated with multiple organ failure and mortality of sepsis [25], but pharmaceutical agents targeting endotoxins, such as anti-endotoxin monoclonal antibody, Toll-like receptor 4 antagonist, and endotoxin adsorption using polymyxin B, have shown negative results in clinical trials [26,27,28]. The results of these studies suggest that the endotoxin is just a trigger of the multiple organ failure and mortality of sepsis, and host components may play essential roles. Previous studies have established that the auto-amplification of cytokine expression during sepsis is mediated through the positive feedback between NF-κB–mediated cytokine expression and cytokine-induced activation of the NF-κB pathway [29]. Our finding showed that this positive feedback can be modulated by Wnt signaling in multiple organs during sepsis: We found the interaction between Wnt and NF-κB signaling in multiple organs of endotoxemic mice. This interaction is mediated by the protein–protein interaction between β-catenin and NF-κB, evidenced by co-localization (Figure 5) and co-immunoprecipitation (Figure S6). We also found that a Wnt signaling inhibitor Wnt-C59 inhibits NF-κB signaling. Our results suggest that the activation of NF-κB signaling during sepsis may need functional Wnt signaling to provide β-catenin for its interaction with NF-κB. The reduction of β-catenin by Wnt-C59 leads to the decrease in NF-κB activity and cytokine production, disrupting the positive feedback of NF-κB signaling and cytokine expression in multiple organs during sepsis. We found that Wnt signaling is activated in multiple organs during sepsis, and a Wnt signaling inhibitor Wnt-C59 inhibits the cytokine storm and multiple organ damage. Wnt-C59 does not directly block NF-κB signaling but inhibits NF-κB-mediated cytokine expression via reducing the interaction between β-catenin and NF-κB. Our findings suggest the molecular mechanism, at least partially, underlying the cytokine storm and multiple-organ dysfunction in sepsis, and also suggests that Wnt signaling in the multiple organs can be a therapeutic target of sepsis. The results of our study are supported by the results of previous studies reporting that β-catenin overexpression induces NF-κB activation in rat cardiomyocytes [30], and knocking down β-catenin reduces NF-κB activity in human bronchial epithelial cells [31]. It should be taken into account, however, that the interaction of β-catenin with NF-κB is context-dependent, and the effect of this interaction varies according to the type of the cell, tissue, or stimulus [32].

In the present study, we analyzed the kidney, lung, and liver during endotoxemia; thus, other important organs, including the heart, spleen, those of the central nervous system, and hematological system, need to be analyzed in the future. Most experiments of this study were conducted at 6 h after LPS injection, but the time course experiments should be conducted to analyze the expression of cytokines and signal transducing proteins in various time points after induction of endotoxemia. This study used endotoxemic mice, induced by LPS or *E. coli*, as an animal model of sepsis, but the animal model generated via cecal ligation and puncture, which is a gold standard animal model of sepsis, needs to be assessed as well. Additionally, the effects of diverse Wnt-signaling inhibitors, as well as the knocking down/out of various Wnt-pathway genes, remain to be analyzed.

In this study, we focused on proinflammatory cytokine upregulation, but other components of sepsis need to be investigated in multiple organs, such as the activation of the complement system, dysfunction of the barrier system, and modulation of metabolism [33]. Recent studies reported that the complement system may play important roles in sepsis mortality and organ damage: The anti-C5 antibody reduced mortality, bacterial load, and lung injury [34]. C6 knockout mice showed improved survival, reduced levels of proinflammatory cytokines, and reduced lung injury during sepsis [35]. It should also be noted that some studies suggested the relation of a complement system with Wnt/β-catenin or NF-κB signaling. Complement C3a was reported to activate Wnt/β-catenin signaling and induce brain white matter damage in a neonatal septic rat induced by LPS [36]. C1a was reported to activate Wnt/β-catenin signaling by binding to Frizzled receptors [37]. NF-κB is involved in the expression of many complement proteins, including C3 and C4 [38,39]. More studies are necessary for the role of Wnt signaling and its interaction with NF-κB signaling in complement activation during sepsis along with the effects of Wnt signaling inhibitors.

## 4. Materials and Methods

### 4.1. Animals and Reagents

Male 4-week-old C57BL/6 mice (18–20 g), purchased from Raon Bio Inc. (Yongin, Korea), were housed with a 12 h/12 h light/dark cycle in the animal facility of the Chung-Ang University (Seoul, Korea) and fed a standard laboratory diet. They were acclimated to the environment for 7 d before they were used in experiments. LPS from *Klebsiella pneumoniae* was obtained from Sigma-Aldrich (St Louis, MO, USA). *Escherichia coli* (*E. coli*) K12 strain was obtained from American Type Culture Collection (Manassas, VA, USA). Wnt-C59 was purchased from Med Chem Express (Monmouth Junction, NJ, USA).

### 4.2. Animal Experiments

The animal-research protocol for this study was approved by the institutional animal care and use committee in Chung-Ang University (Approval Number 2019-00064). Wnt-C59 was dissolved in saline and intraperitoneally (i. p.) injected at various doses 2 h before an i. p. injection of 25 mg/kg of LPS. The optimum dose of LPS was determined through preliminary experiments. Saline-injected mice were used as a control group. At 6 h after LPS injection, the mice were anesthetized using 10 mg/kg of alfaxalone (Jurox Inc., North Kansas City, MO, USA). The kidneys, lungs, and liver of each mouse were collected, rapidly frozen in liquid nitrogen, and then stored at −70 °C until needed. Whole blood was collected into ethylenediaminetetraacetic-acid–coated tubes, followed by centrifugation to collect the plasma. The concentrations of cytokines (e.g., TNF-α, IL-6, IL-1β, IL-1α, MCP-1, and RANTES) in the plasma were measured using a Luminex assay (R&D Systems, Inc., Minneapolis, MN, USA). The levels of blood urea nitrogen (BUN), alanine aminotransferase (ALT), and aspartate aminotransferase (AST) in the plasma samples were quantified using a veterinary biochemistry analyzer (Samsung, Suwon, Korea).

### 4.3. Measurement of Cytokine mRNA Levels and NF-κB Target-DNA Binding Activity

Total RNA was extracted from the kidney, lung, and liver by using the RNeasy kit (Qiagen, Hilden, Germany). RT-qPCR was performed as previously described [40]. Assay-on-Demand Gene Expression Products (Applied Biosystems Inc., Foster City, CA, USA) were used for RT-qPCR to measure the *TNF-α* (Cat. No. Mm00443258_m1), interleukin (*IL*)-6 (Cat. No. Mm00446190_m1), and *IL-1β* (Cat. No. Mm00434228_m1) mRNA and 18S ribosomal RNA (Cat. No. Hs99999901_s1) levels in the kidney, lung, and liver of endotoxemic mice. For each sample, the mRNA levels were normalized against the corresponding 18S ribosomal RNA level, and the normalized mRNA levels in each group were compared with those in a control group by using the comparative Ct method [41]. The target-DNA binding activity of NF-κB was quantified using TransAM NF-κB ELISA (Active Motif, Carlsbad, CA, USA), as described previously [40].

### 4.4. Western Blotting

Kidney, lung, and liver extracts were prepared using the NucleoSpin kit (Macherey-Nagel, Duren, Germany). Western blotting was conducted as previously described [40]. Anti-IκB (diluted 1:1000), anti-phospo-IκB (Ser 32/36) (1:1000), anti-NF-κB p65 (1:1000), anti-LRP6 (1:1000), anti-phospho-LRP6 (Ser 1490) (1: -500), anti-Axin (1:1000), and anti-phospho-β-catenin (Ser 33/37) (1:2000) primary antibodies; and anti-mouse (1:1000) and anti-rabbit (1:1000) secondary antibodies were from Cell Signaling Technologies Inc. (Beverly, MA, USA). The anti-β-catenin (1:1000) antibody was from BD Transduction Laboratories Inc. (Lexington, KY, USA). Anti-β-actin (1:1000) and anti-TBP (TATA-box–binding protein) (1:1000) antibodies were from Santa Cruz Biotechnology Inc. (Santa Cruz, CA, USA).

### 4.5. Immunofluorescence Microscopy and Co-Localization Assay

The kidneys, lungs, and liver of each mouse were rapidly frozen in liquid nitrogen and embedded in OCT compound (Sakura, Tokyo, Japan). The embedded organ samples were sectioned at 10 μm thickness. The organ sections were then fixed with cold acetone for 10 min, followed by incubation in a blocking buffer (CAS Block, Thermo Fisher Scientific, Wilmington, DE, USA) at 25 °C for 1 h. Subsequently, they were incubated at 4 °C overnight with mouse anti-β-catenin (1:200) and rabbit anti-NF-κB (1:200) antibodies in phosphate-buffered saline (PBS). Next, the sections were washed three times with PBS containing 0.03% Triton X-100 and then incubated with Cy3-labeled donkey anti-rabbit IgG (1:400; Jackson ImmunoResearch Laboratories, Inc., West Grove, PA, USA) and FITC-labeled donkey anti-mouse IgG (1:400; Jackson ImmunoResearch Laboratories) antibodies in PBS at 25 °C for 1 h. After counterstaining with 1 μg/mL of DAPI (Sigma-Aldrich), the sections were examined using a confocal microscope (Nikon, Tokyo, Japan).

### 4.6. Statistical Analyses

All data are presented as the mean ± standard deviation of at least three replicate experiments. Statistically significant differences between experimental groups were detected using the unpaired *t*-test, and *p*-values < 0.05 were considered significant. All statistical analyses were performed using SPSS ver. 14 (SPSS, Chicago, IL, USA).

## Data Availability

The data presented in this study are available on request from the corresponding author.

## Supplementary Materials

The following are available online at https://www.mdpi.com/article/10.3390/ijms22126249/s1, Figure S1: Wnt-C59 downregulated the *MCP-1* and *IL-1α* mRNA levels in multiple organs of endotoxemic mice. Figure S2: Wnt-C59 suppressed the NF-κB and Wnt/β-catenin pathways in the lung of endotoxemic mice. Figure S3: Wnt-C59 suppressed the NF-κB and Wnt/β-catenin pathways in the liver of endotoxemic mice. Figure S4: Immunofluorescence analysis of the lung sections from endotoxemic mice for the major proteins in the Wnt/β-catenin pathway. Figure S5: Immunofluorescence analysis of the liver sections from endotoxemic mice for the major proteins in the Wnt/β-catenin pathway. Figure S6: Wnt-C59 inhibited the co-immunoprecipitation of β-catenin and NF-κB in multiple organs of endotoxemic mice.
