# Supplementary material for: Wnt-Signaling Inhibitor Wnt-C59 Suppresses the Cytokine Upregulation in Multiple Organs of Lipopolysaccharide-Induced Endotoxemic Mice via Reducing the Interaction between β-Catenin and NF-κB
Source: Int J Mol Sci. 2021 Jun 10;22(12):6249. doi: 10.3390/ijms22126249 (PMC8230366; doi:10.3390/ijms22126249)
Supplement: Supplementary file 1 [file ijms-22-06249-s001.zip › ijms-1239481-supplementary.pdf]

# Supplementary Data

**The Wnt-signaling inhibitor Wnt-C59 suppresses the cytokine upregulation in multiple organs of lipopolysaccharide-induced endotoxemic mice via reducing the interaction between β-catenin and NF-κB**

Jaewoong Jang[a, 1], Jaewon Song[a, 1], Inae Sim[a], Young V. Kwon[b], and Yoosik Yoon[a, *]

[a] Department of Microbiology, Chung-Ang University College of Medicine, Seoul 06974, Republic of Korea

[b] Department of Biochemistry, University of Washington, Seattle, WA 98195, USA

[1] Co-first authors

*Corresponding author:

Yoosik Yoon

Department of Microbiology, Chung-Ang University College of Medicine

Seoul 06974 Republic of Korea

Tel.: +82-2-820-5767, Fax: +82-2-813-5387, Email address: thanks@cau.ac.kr

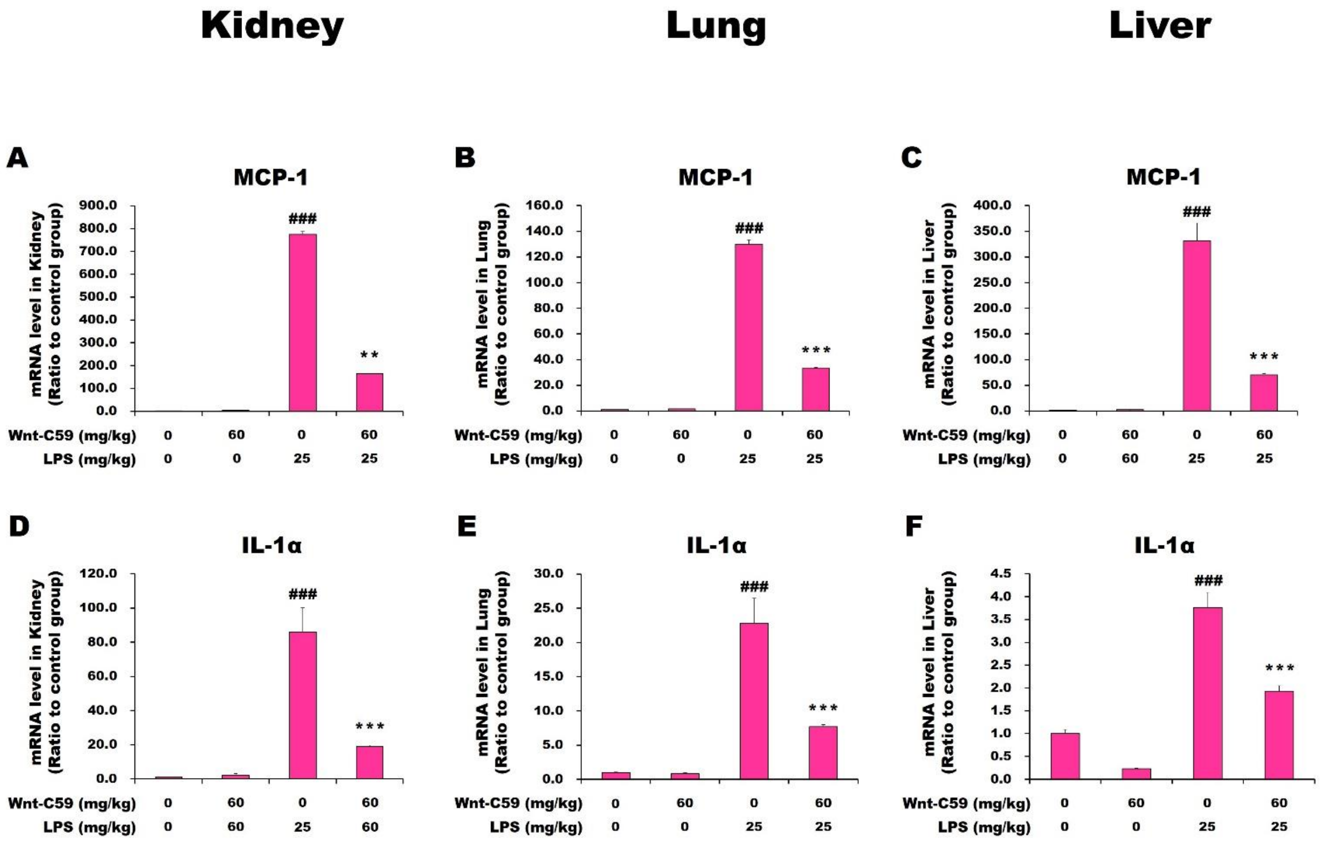


**Figure S1. Wnt-C59 downregulated the *MCP-1* and *IL-1α* mRNA levels in multiple organs of endotoxemic mice.**

C57BL/6 mice were i. p. injected with 0 or 60 mg/kg Wnt-C59 and 0 or 25 mg/kg lipopolysaccharide (LPS). The *MCP-1* and *IL-1α* mRNA levels in the kidney, lung, and liver were quantified using reverse transcription–polymerase chain reaction. Data show mean ± standard deviation (n = 4). $**P < 0.01$, $***P < 0.001$ compared with the group injected with 25 mg/kg LPS. $\#\#\#P < 0.001$ compared with the contr ol group (unpaired *t*-test).

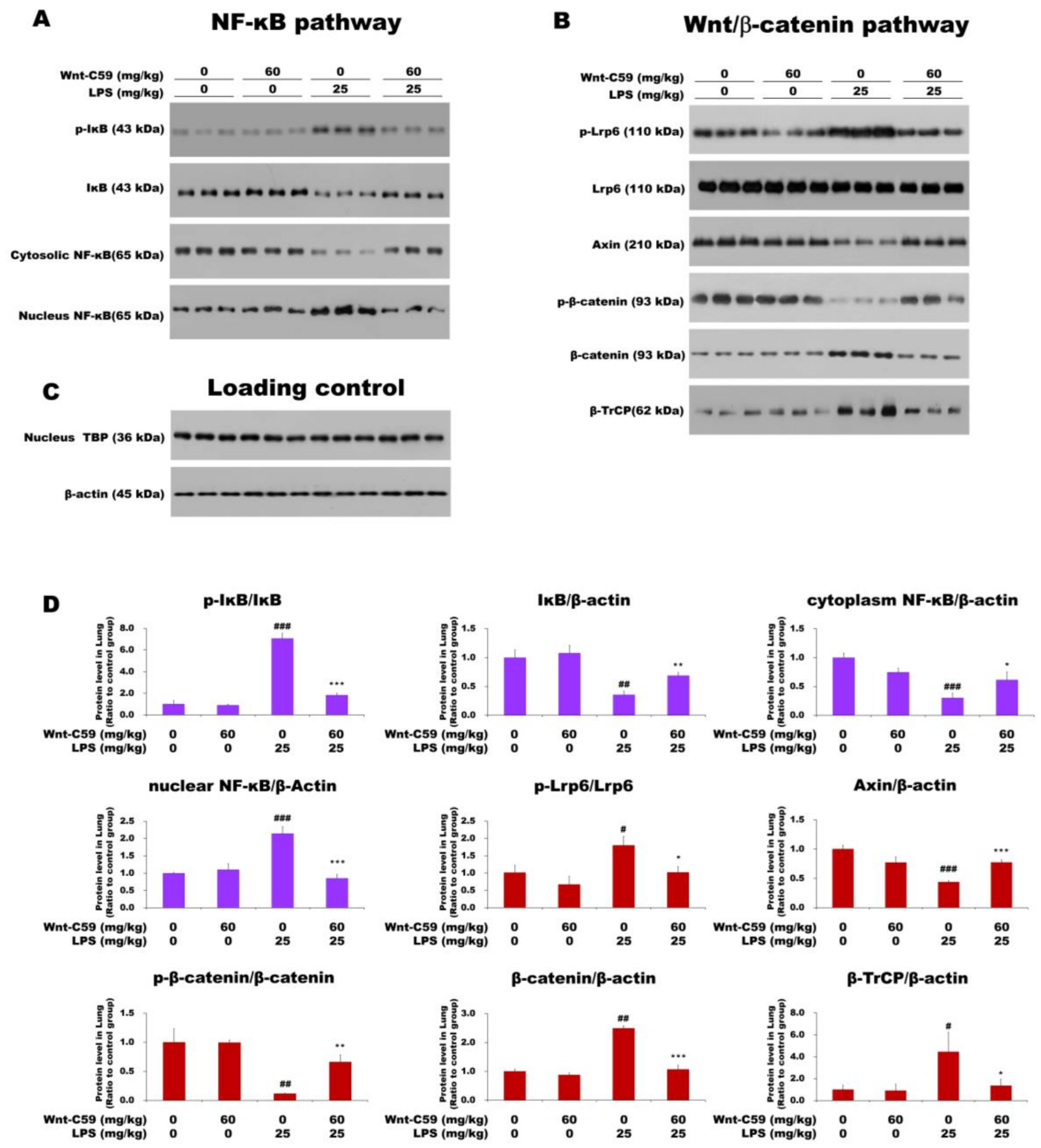


**Figure S2. Wnt-C59 suppressed the NF-κB and Wnt/β-catenin pathways in the lung of endotoxemic mice.** C57BL/6 mice were i. p. injected with 0 or 60 mg/kg Wnt-C59 and 0 or 25 mg/kg lipopolysacc haride (LPS). To measure the levels of the proteins involved in the NF-κB (A) and Wnt/β-catenin (B) pathways, western blotting was conducted using lung protein extract from the endotoxemic mice (n = 3). (C) β-Actin and TBP were used as loading controls for total and nuclear lysates, respectively. (D) The western-blot band intensities of the members of the NF-κB and Wnt/β-catenin pathways are shown in violet and red, respectively. The target band intensities were quantified using ImageJ (NIH, Bethesda, MD, USA) and normalized to the band instensities of the loading controls. Data show average ± standard deviation (n = 3). *$P < 0.05$, **$P < 0.01$, ***$P < 0.001$ compared with the group injected with 25 mg/kg LPS. #$P < 0.05$, ##$P < 0.01$, ###$P < 0.001$ compared with the control group (unpaired *t*-test). TBP: TATA-box–binding protein

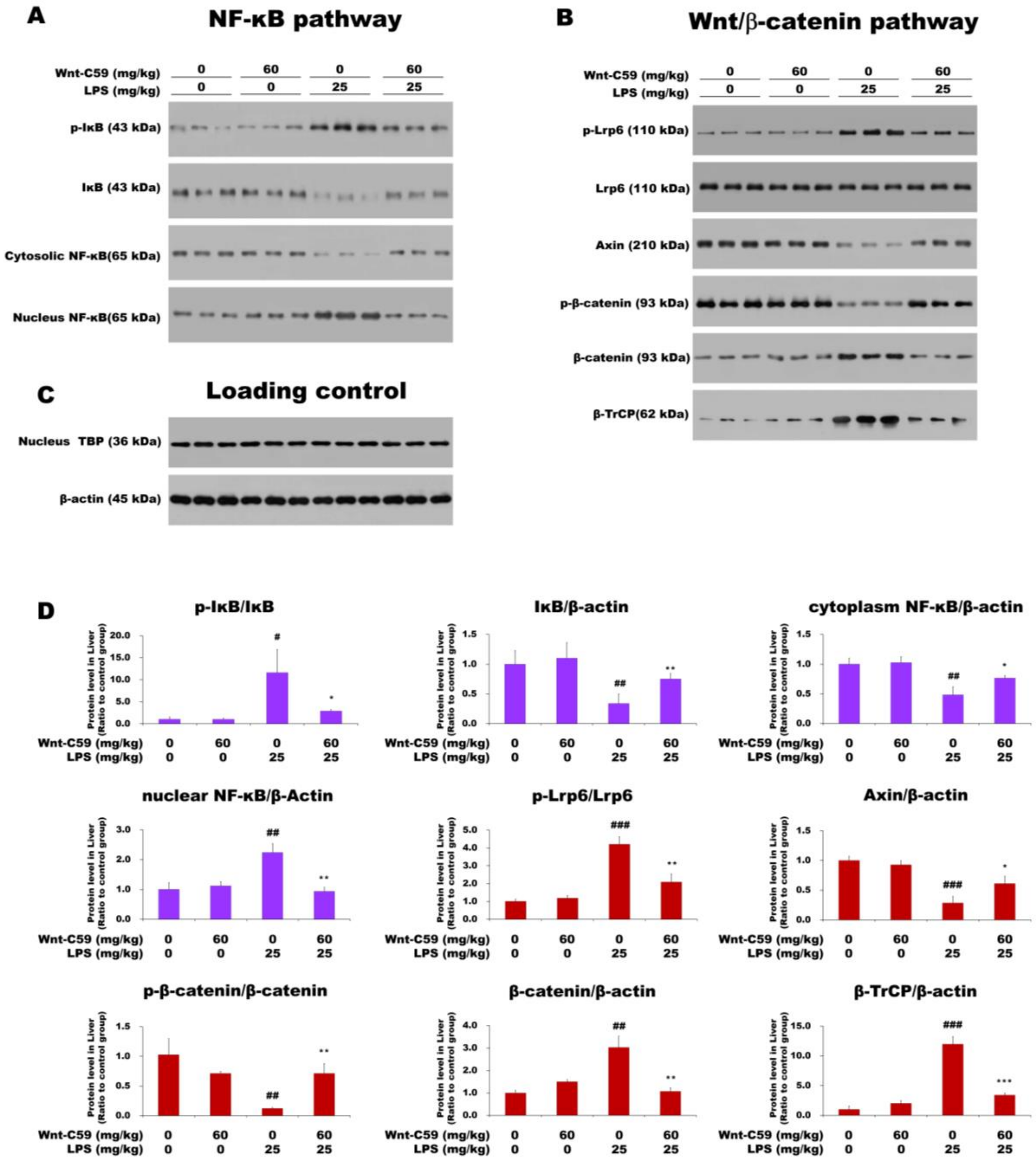


**Figure S3. Wnt-C59 suppressed the NF-κB and Wnt/β-catenin pathways in the liver of endotoxemic mice.** C57BL/6 mice were i. p. injected with 0 or 60 mg/kg Wnt-C59 and 0 or 25 mg/kg lipopolysacc haride (LPS). To measure the levels of the proteins involved in the NF-κB (A) and Wnt/β-catenin (B) pathways, western blotting was conducted using liver protein extract from the endotoxemic mice (n = 3). (C) β-Actin and TBP were used as loading controls for total and nuclear lysates, respectively. (D) The western-blot band intensities of the members of the NF-κB and Wnt/β-catenin pathways are shown in violet and red, respectively. The target-band intensities were quantified using ImageJ (NIH, Bethesda, MD, USA) and normalized to the band intensities of the loading controls. Data show average ± standard deviation (n = 3). **P* < 0.05, ***P* < 0.01, ****P* < 0.001 compared with the group injected with 25 mg/kg LPS. #*P* < 0.05, ##*P* < 0.01, ###*P* < 0.001 compared with the control group (unpaired *t*-test). TBP: TATA-box–binding protein

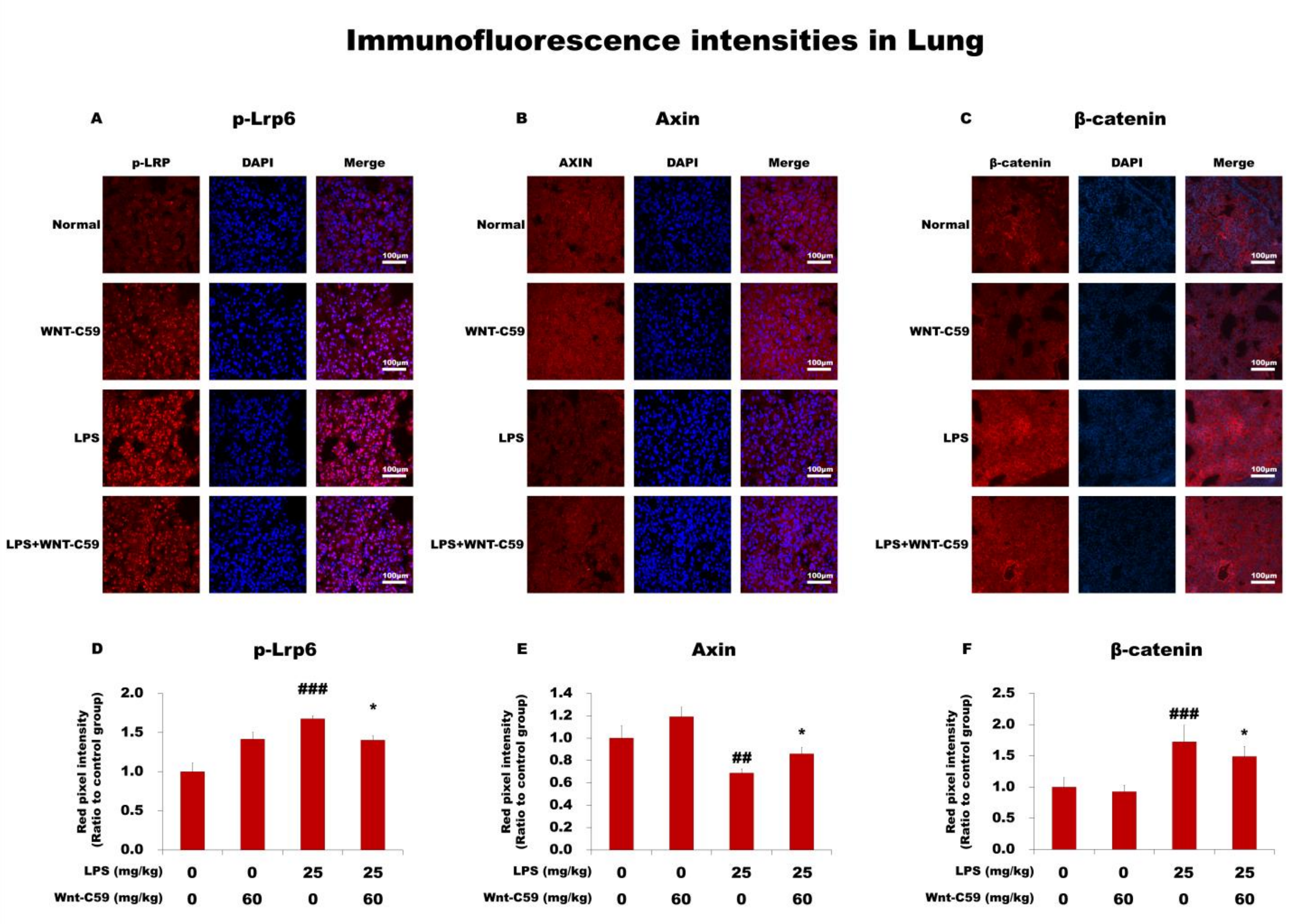


**Figure S4. Immunofluorescence analysis of the lung sections from endotoxemic mice for the major proteins in the Wnt/β-catenin pathway.**

The fluorescence signals in the confocal microscopic images were analyzed using ImageJ (NIH, Bethesda, MD, USA). C57BL/6 mice were i. p. injected with 0 or 60 mg/kg Wnt-C59 and 0 or 25 mg/kg lipopolysaccharide (LPS). *$P < 0.05$ compared with the group injected with 25 mg/kg LPS. ##$P < 0.01$, ###$P < 0.001$ compared with the control group (unpaired *t*-test).

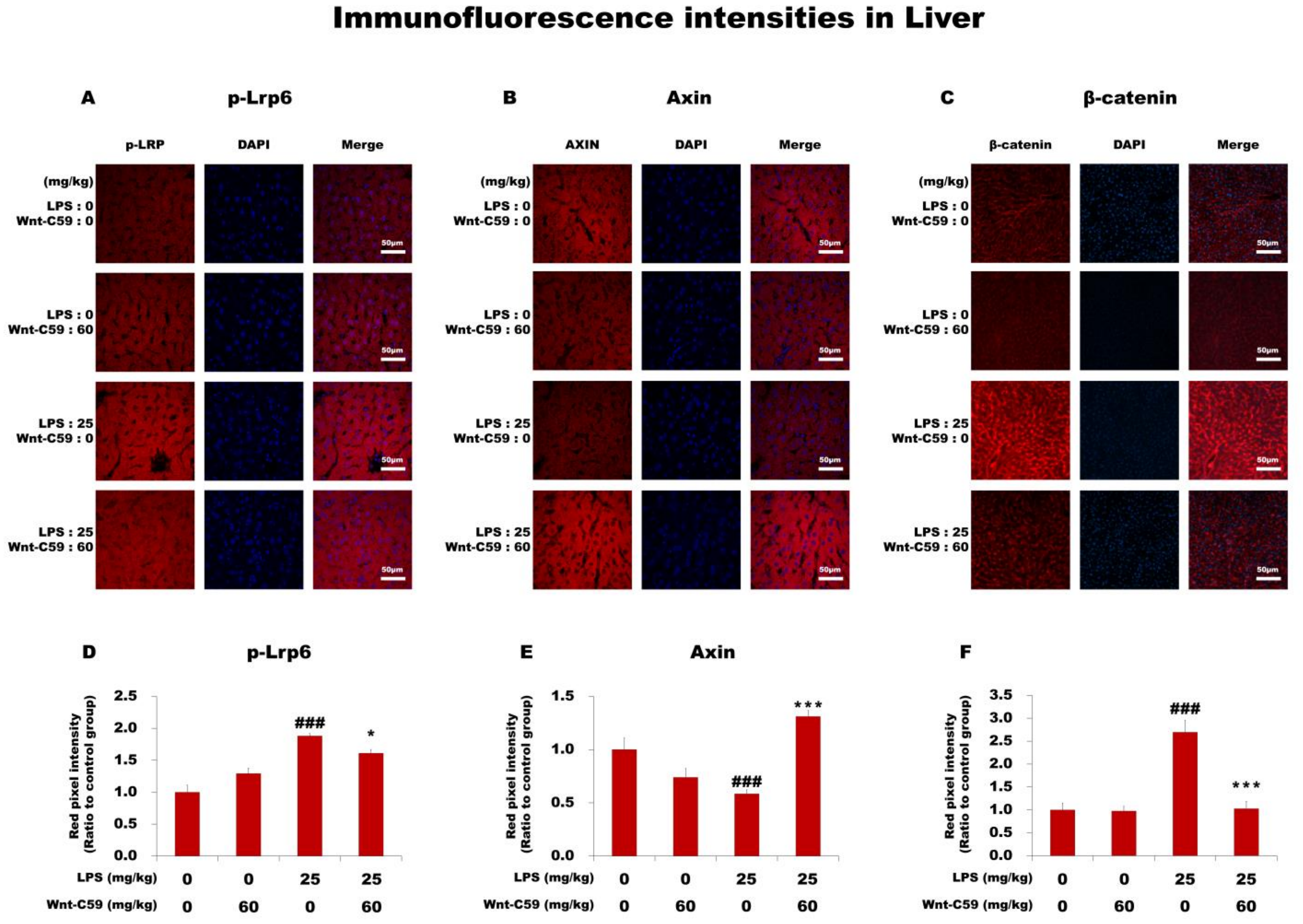


**Figure S5. Immunofluorescence analysis of the liver sections from endotoxemic mice for major proteins in the Wnt/β-catenin pathway.**

The fluorescence signals in the confocal microscopic images were analyzed using ImageJ (NIH, Bethesda, MD, USA). C57BL/6 mice were i. p. injected with 0 or 60 mg/kg Wnt-C59 and 0 or 25 mg/kg lipopolysaccharide (LPS). *$P < 0.05$, ***$P < 0.001$ compared with the group injected with 25 mg/kg LPS. ###$P < 0.001$ compared with the control group (unpaired *t*-test).

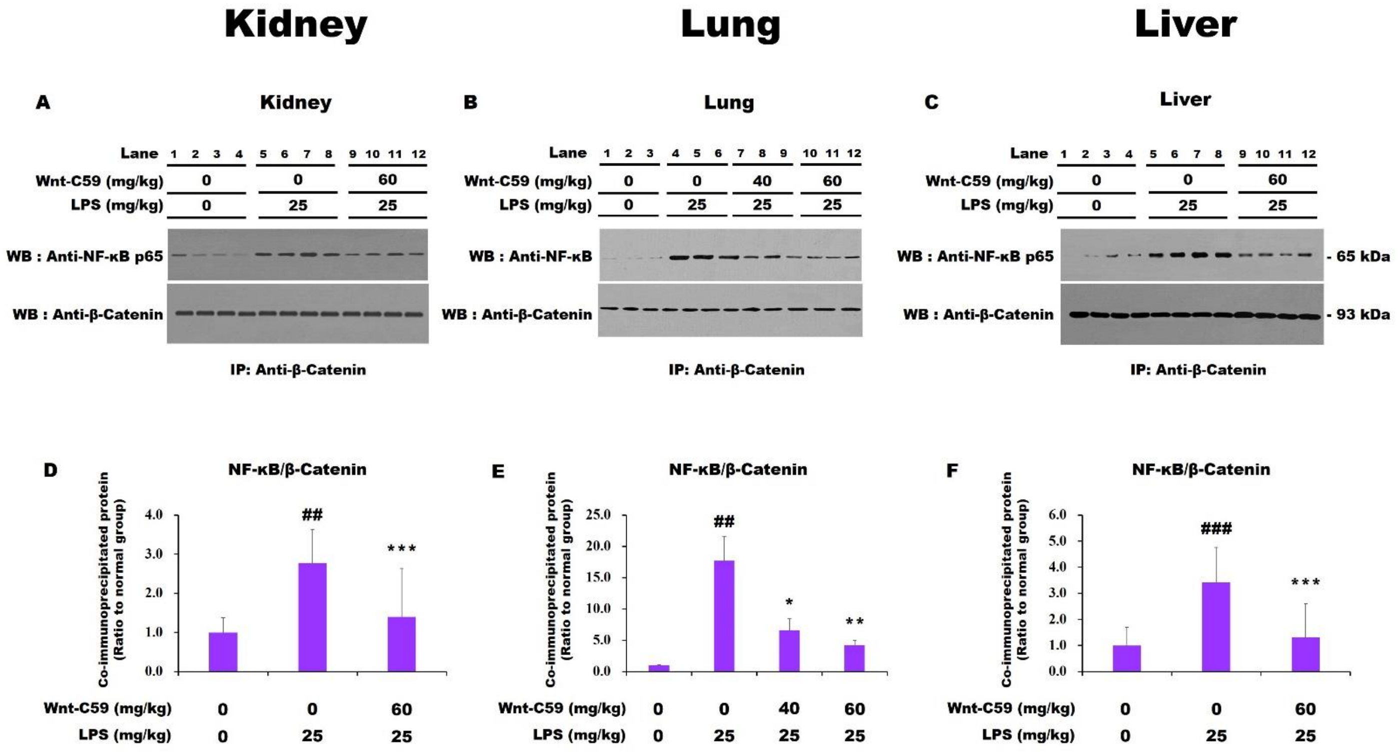


**Figure S6. Wnt-C59 inhibited the co-immunoprecipitation of β-catenin and NF-κB in multiple organs of endotoxemic mice.**

C57BL/6 mice were i. p. injected with 0 to 60 mg/kg Wnt-C59 and 0 or 25 mg/kg of lipopolysaccharide (LPS). Co-immunoprecipitation experiments were performed using an immunoprecipitation kit from BioVision (Mountain View, CA, USA) with kidney, lung, and liver extracts. The amount of NF-κB bound to β-catenin was measured via western blotting with an anti-NF-κB antibody after immunoprecipitation with an anti–β-catenin antibody. Band intensities were quantified using ImageJ (NIH, Bethesda, MD, USA). Data show mean ± standard deviation (n = 3 or 4). **P* < 0.05, ***P* < 0.01, ****P* < 0.001 compared with the group injected with 25 mg/kg LPS. ##*P* < 0.01, ###*P* < 0.001 compared with the control group (unpaired *t*-test). WB: western blotting, IP: immunoprecipitation.
